# Application of a Sensitive and Reproducible Label-Free Proteomic Approach to Explore the Proteome of Individual Meiotic-Phase Barley Anthers

**DOI:** 10.3389/fpls.2019.00393

**Published:** 2019-04-02

**Authors:** Dominika Lewandowska, Runxuan Zhang, Isabelle Colas, Nicola Uzrek, Robbie Waugh

**Affiliations:** ^1^Cell and Molecular Sciences, The James Hutton Institute, Dundee, United Kingdom; ^2^Information and Computational Sciences, The James Hutton Institute, Dundee, United Kingdom; ^3^School of Life Sciences, University of Dundee, Dundee, United Kingdom

**Keywords:** label-free micro-proteomics, plants, meiosis, anthers, meiocytes, mass spectrometry LC-MS/MS

## Abstract

Meiosis is a highly dynamic and precisely regulated process of cell division, leading to the production of haploid gametes from one diploid parental cell. In the crop plant barley (*Hordeum vulgare*), male meiosis occurs in anthers, in specialized cells called meiocytes. Barley meiotic tissue is scarce and not easily accessible, making meiosis study a challenging task. We describe here a new micro-proteomics workflow that allows sensitive and reproducible genome-wide label-free proteomic analysis of individual staged barley anthers. This micro-proteomic approach detects more than 4,000 proteins from such small amounts of material as two individual anthers, covering a dynamic range of protein relative abundance levels across five orders of magnitude. We applied our micro-proteomics workflow to investigate the proteome of the developing barley anther containing pollen mother cells in the early stages of meiosis and we successfully identified 57 known and putative meiosis-related proteins. Meiotic proteins identified in our study were found to be key players of many steps and processes in early prophase such as: chromosome condensation, synapsis, DNA double-strand breaks or crossover formation. Considering the small amount of starting material, this work demonstrates an important technological advance in plant proteomics and can be applied for proteomic examination of many size-limited plant specimens. Moreover, it is the first insight into the proteome of individual barley anther at early meiosis. The proteomic data have been deposited to the ProteomeXchange with the accession number PXD010887.

## Introduction

Proteomics is a potent approach to identify and explore functionally important mechanisms and processes in diverse fields of biology. It has been enabled by recent and significant improvements in Mass Spectrometry (MS) technology and instrumentation sensitivity that have resulted in increased sequence coverage and the total number of proteins detected in a typical proteomics experiment, including low abundant proteins. When used appropriately, both so-called global and targeted proteomic strategies have been used effectively many times for protein, gene and network identification in biological systems ranging from yeast (Hebert et al., 2014) to mammals (Geiger et al., 2012). By comparison, proteomics studies have been relatively infrequently used in plant biology. When they have been used successfully, global proteome studies have revealed a repertoire of up to 13,029 proteins in Arabidopsis by merging information from multiple tissue and organ samples (Baerenfaller et al., 2008) and, as an example, in targeted studies over 500 ubiquitylated proteins from different leaf tissue cells were identified after inhibition of the Ubiquitin-26S proteasome with the specific inhibitor Syringolin A followed by affinity enrichment of ubiquitinylated proteins (Svozil et al., 2014, 2015). In these cases, protein identification was greatly facilitated by the quality of the Arabidopsis genome sequence and its associated annotation. Now, with increasing amounts of high-quality crop genome information, genome-wide proteomic studies are also being successfully conducted in crop plants (e.g., Szymanski et al., 2017). A common feature of these is that they generally use relatively abundant starting biological materials. Thus, 11,552 proteins were identified in different tissues of the maize primary root (Marcon et al., 2015) as were 8,588 proteins from the proteome of tomato pericarp tissues at the ripe red stage (Mata et al., 2017). While these represent significant successes, a major challenge that remains is how to best exploit the available and increasingly powerful proteomics technologies to understand cell and tissue specific processes or to identify and interrogate dysregulated cellular networks induced by variables that include developmental or environmental change, or genetic mutation (genotype).

A potentially powerful situation would be to study individual tissues or cell types that change over time or are perturbed by biotic or abiotic stimuli. However, studying the proteome of a specific tissues or single cell can be difficult due to the biological starting materials often being in short supply. To circumvent this problem for genome-wide cell or tissue specific proteomics studies, enrichment technologies such as microfluidic cell sorting (Kasuga et al., 2017), laser capture microdissection (LCM, Clair et al., 2016), sub-cellular organelle purification (Lee et al., 2012; Kamal et al., 2013) and transgenic cell labeling strategies such as INTACT (Deal and Henikoff, 2011) have offered possible solutions. Unfortunately, in plants at least, they have generally met with limited success in terms of numbers of proteins identified, a typical experiment generally revealing only tens to hundreds of proteins. This output can often be attributed to the abundance of the substrate, methods used for protein extraction and preparation, the methodology used for detection and sensitivity of available instruments. Recently Bensaddek et al. (2016) addressed the sample abundance issue by developing a powerful label-free genome-wide micro-scale approach for shotgun proteomics analysis at the level of a single *Caenorhabditis elegans* worm (∼1–1.2 mm long). These authors were able to detect ∼3,000 proteins across a wide dynamic range (over 6 orders of magnitude). They subsequently used the approach to investigate the *C. elegans* proteome in response to the application of heat shock at a single worm level.

We are attempting to understand and manipulate the characteristic patterns of recombination that occur during meiosis in the crop plant barley as a means toward releasing genetic variation currently locked in the large non-recombining but gene rich pericentromeric regions of each of its seven large chromosomes (Mascher et al., 2017). In barley, male meiosis occurs in anthers which are located within individual single floret (spikelets) on the developing inflorescence (or spike) (Mittmann et al., 2018). Each barley spike harbors around 20–40 individual spikelets arranged alternatively either side of a central axis or rachis. Inside each spikelet three developmentally synchronized anthers together produce hundreds of male gametes in the form of pollen grains, along with a single female gametic cell that upon fertilization will develop into a single barley seed. Meiosis occurs over a restricted period of around 48 h in barley with recombination occurring in the earlier stages within the first 12–24 h (Higgins et al., 2012). Understandably, most temporally refined studies of meiosis and recombination in plants routinely use immuno-cytological investigation of the developing meiocytes, which provides a relatively abundant supply of developmentally staged meiotic cells. A recent targeted affinity proteomics study in *Brassica oleracea* showed the network of proteins interacting with the meiotic chromosome axis protein, ASY1 (Osman et al., 2018). For genome wide molecular studies, pooled populations of pollen mother cells have been employed previously (Collado-Romero et al., 2014; Ye et al., 2015, 2016; Li et al., 2018) but temporal, and to a degree spatial, resolution is unavoidably compromised.

To study the rapid and highly dynamic developmental process of meiosis at the molecular level we need to be able to identify accurately when meiosis occurs and the precise meiotic stage of any sampled biological materials. In barley the availability of multiple developmentally synchronized anthers per spikelet provides a unique opportunity to accurately stage meiosis by sacrificing one, while leaving the remainder, which are effectively biological replicates, for molecular studies (Mittmann et al., 2018). Meiosis typically occurs in anthers of 6 to 7-week-old barley plants when the entire inflorescence is still buried deep within the leaf sheathes on the developing tiller (stem) and is less than 1.5 cm long. Consequently, developing meiotic phase anther tissues are small. Within the inflorescence the torpedo-shaped anthers range from around 0.4–0.9 mm in length and sample availability and abundance is therefore low (Gómez and Wilson, 2012). Therefore, there are some obvious parallels between our objectives and the recent micro-proteomics study in *C. elegans* (ref above). Where it is possible to reliably and reproducibly assay protein representation and abundance in single meiotically staged anthers, the benefits of biological resolution, accurate replication and ease of sampling could by far outweigh the alternative of bulk tissue collection with the inevitable blurring of developmental transitions and subsequent analysis and interpretation.

For both practical and biological reasons, we were keen to explore the possibility of performing shotgun label-free proteomics on small numbers of isolated and meiotically staged barley anthers, and possibly individual populations of meiocytes. Here we describe the first phase results of these investigations as a pipeline that allows sensitive and reproducible genome-wide label-free proteomic analysis of 0.5–0.6 mm staged barley anthers (*Hordeum vulgare* cv. Golden Promise) containing meiocytes at early stages of meiosis. We compared the results obtained from one and two anthers from a single spikelet, to 5 anthers from a pair of adjacent spikelets, alongside results from a macro-proteomic study obtained from sampling over 1,000 individual anthers. We show that our micro-proteomic pipeline yields highly correlated data across all samples, despite the small number of the starting material, and generally performs as well as our macro-proteomic approach. Importantly our data has the power to reveal new information about the barley meiocyte proteome and the dynamic of the meiotic process along the spikes.

## Materials and Methods

### Chemicals and Reagents

Complete protease inhibitor cocktail tablets (Cat No: 5892970001) were obtained from Roche. Bicinchoninic Acid Assay (BCA) Kit (Cat No: 23227) was from Pierce. InstantBlue staining kit was obtained from Expedeon (Cat No: ISB). NuPAGE minigels (Cat No: NP0321BOX), LDS sample buffer (Cat No: NP0007) and NuPAGE MOPS (Cat No: NP0001) and MES running buffers (Cat No: NP0002) were from Thermo Fisher. Trypsin was obtained from Promega (Cat No: 90058). C18 cleaning columns were from Applied Biosystems (Cat No: 1112906) and the Pepmap C18 columns were from Dionex (Cat No: 160321). All other reagents were obtained from Sigma.

### Plant Material

We used barley (*H. vulgare*) cv. Golden Promise. The seeds were obtained from The James Hutton Institute seed store. Plants were grown in 70% humidity under 16 h of light at 18–20°C and 8 h of dark at 16°C until they reached meiosis (6–7 weeks) in a controlled environment growth room. For anthers collection, 0.8–1.4 cm spikes (i.e., developing barley inflorescences) were collected and 0.6 mm anthers dissected manually with insulin syringes on a plastic Petri dish under a stereoscopic microscope (with graticule). Each barley spikelet contains three developmentally synchronized anthers. Meiosis was monitored in one anther from each spikelet by squashing, staining with a solution of 2% acetocarmine and observation under a Microtec light microscope according to Colas et al. (2016). The remaining two anthers were retained. For micro-proteomics single 0.6 mm anthers were dissected, placed on a cavity glass slide in a drop of 1 × PBS including protease inhibitor cocktail prepared according to the manufacturer’s instructions, and squashed with an insulin needle. Samples were placed immediately in an Eppendorf tube containing LDS NuPage buffer including one tenth volume of NuPAGE sample reducing agent. For the micro-proteomics approach three biological replicates were used.

For the macro-proteomic experiment approximately 1,000 staged anthers at the leptotene/zygotene stage of meiosis (within a size range of 0.6–0.8 mm) were collected, immediately frozen in liquid nitrogen and stored at -20°C until use. These were subsequently sub-divided into seven biological replicates, each consisting of approximately 140 anthers.

### Preparation of Protein Extracts for Macro-Proteomic Analysis

Frozen barley anthers were suspended in extraction buffer [50 mM Tris HCl pH 7.6, 0.33 M sucrose, 1 mM MgCl2, 1 mM DTT, 1% (w/v) C7BzO, and protease inhibitor cocktail], placed in glass embryo dish and meiocytes released by crushing anthers with a glass rod. Meiocyte enriched samples were collected with an insulin syringe into a fresh tube and sonicated 3 × 30 s, with an interval of 1 min between each cycle, at 4°C using Bioruptor sonicator (Diagenode). Then, samples were extracted for 45 min on ice and the lysates were centrifuged for 10 min at 4,000 *g* at 4°C. Supernatants were collected in fresh tubes and centrifuged again for 10 min at 16,000 *g* at 4°C. Supernatants were collected in fresh tubes and pellets containing insoluble proteins extracted again with 20 μL of extraction buffer for 45 min on ice. Supernatants from both extractions were then pooled. A Bicinchoninic Acid Assay (BCA) was performed on the supernatants to determine protein concentration.

### Sample Preparation by 1D SDS/PAGE Gel Fractionation

For micro-proteomics, individual squashed anthers were put into Eppendorf tube, extracted with the LDS NuPage buffer. Size fractionation of protein extracts was achieved by quick SDS-PAGE analysis on 4–12% (w/v) Bis-Tris NuPage gels (Thermo) using MES running buffer (50 mM MES, 50 mM Tris Base, 0.1% SDS, 1 mM EDTA, pH 7.3). Electrophoresis was run for only 10–15 min at 200 V constant. Gels were stained with InstantBlue according to manufacturer’s instructions (Expedeon). Each gel track was cut into 3 fractions, corresponding to 3–17, 17–62, and 62–250 kDa. Gel pieces were de-stained by immersion in 50% acetonitrile and proteins reduced by incubation with 10 mM DTT in 20 mM Ammonium Bicarbonate and alkylated with 50 mM iodoacetamide in 20 mM Ammonium Bicarbonate by incubating for 30 min in the dark. The gel slices were then digested with trypsin 12.5 μg/mL in 20 mM Ammonium Bicarbonate overnight at 30°C, shaking at 300 rpm on an Eppendorf Thermomixer shaker and peptides were extracted the next day by incubating the gel pieces in 50% acetonitrile. Samples were dried to approximately 10 μL by vacuum centrifugation in an Eppendorf speedvac at room temperature.

For Macro-proteomics, size fractionation of the combined proteins was achieved by SDS-PAGE analysis, as above. A maximum of 25 μg of protein was loaded per lane and the electrophoresis was run for 35 min at 200 V constant. Each lane from the gel was cut into eight fractions, corresponding to 3–17, 17–28, 28–40, 40–56, 56–73, 73–130, 130–270, and bigger than 270 kDa. Gel staining, destaining, reduction and alkylation was performed as above. The gel slices were double digested with 2 μg/mL trypsin in 50 mM Ammonium Bicarbonate (with one digest done overnight at 37°C, followed by fresh trypsin aliquot addition, 4 h shaking at 37°C). Peptides were extracted as described above then cleaned over an in-house C18 column (POROS R2, Applied Biosystems) as follows: The column was first activated with a mixture of 70% acetonitrile and 0.1% Trifluoro acetic acid (TFA) and then washed with 0.1% TFA. The whole sample was loaded onto the column and washed with 0.1% TFA. Bound peptides were eluted from the column using a solution of 70% acetonitrile and 0.1% TFA. Samples were dried down to approximately 10 μL using vacuum centrifugation as before.

### Single-Pot Solid-Phase-Enhanced Sample Preparation (SP3) and Hydrophilic Interaction Liquid Chromatography (HILIC)

The SP3 protocol was performed according to Hughes et al. (2012) and HILIC according to the instructions of MagReSyn^®^ HILIC^1^.

### LC-MS/MS and MaxQuant Analysis

A Dionex Ultimate 3000 nanoHPLC system was used with 2 μg of peptides injected onto an Acclaim PepMap C18 nano-trap column (Dionex). After washing with a solution of 2% (vol/vol) acetonitrile and 0.1% (vol/vol) formic acid, peptides were resolved on a 150 mm × 75 μm Acclaim PepMap C18 reverse phase analytical column over a 200 min organic gradient with a flow rate of 300 nl min^-1^. The chromatography performed for these samples was as follows. The gradient commenced with 6 min of 95% buffer A (0.1% formic acid)/5% buffer B (80% acetonitrile, 0.08% formic acid), followed by a linear gradient to 35% buffer B over 130 min, then an increase to 98% buffer B for 22 min duration, and completed with a return to 2% buffer B at minute 153 for 17 min. Ions accepted for MS/MS were 2+ and greater. Dynamic exclusion was set to 45 s, and the inclusion mass width for precursor ions was 10 ppm. Peptides were transferred to the mass spectrometer via an Easy-Spray source with temperature set at 50°C and a source voltage of 2.0 kV. Tandem mass spectrometry analysis was carried out on an LTQ-Velos Orbitrap mass spectrometer (Thermo Scientific) using data-dependent acquisition, measuring and sequencing the top 15 ions.

The resulting raw files were processed and searched, using MaxQuant version 1.5.6.5 and the Andromeda peptide search engine (Cox and Mann, 2008; Cox et al., 2011), against the Uniprot Hordeum vulgare database, containing 74,896 protein sequences (March 2017). The database used has been chosen as the one with best sensitivity and specificity of search, after testing Uniprot Oryza sativa, Uniprot Brachypodium distachyon, and Uniprot Hordeum vulgare databases. Enzyme specificity was set as trypsin and the number of missed cleavages permitted was 2. The variable modifications were set as oxidation of methionine and acetylation of the protein N-terminus. Fixed modifications were set to carbamidomethylation of cysteines only. The MS tolerance was set to 7 ppm with the MS/MS tolerance set to 0.5 Da. The peptide and protein False Discovery Rate (FDR) were both set to 1% (Cox and Mann, 2008), and the proteins used for further analysis had 2 or more peptides assigned to them.

The mass spectrometry proteomics data have been deposited to the ProteomeXchange Consortium via the PRIDE partner repository with the dataset identifier PXD010887.

### Protein Function and Gene Ontology Analysis

The output Uniprot identifiers obtained with the MaxQuant software for identified barley proteins were exported to Microsoft Excel for data analysis. FASTA sequences for all proteins were obtained and loaded to Blast2Go Basic v4 for blast analysis against the Spermatophyta protein database. Additionally, Gene ontology (GO) annotations and InterPro domain searches were done to find functional annotations for all barley proteins. Enrichment analysis based on the cellular component and molecular function was performed with AgriGo Singular Enrichment Analysis (SEA) tool^2^ against whole barley Uniprot background. Network interaction analysis was done using the STRING database^3^.

## Results

### Sample Tissues

A consistent feature of many genome-wide proteomic studies is that they typically analyze populations of proteins extracted from tens of thousands to millions of cells derived from complex tissues comprised of multiple cell types, or from bulked small tissue samples isolated from multiple individuals. By default, each of these sampling approaches necessarily mask cell specific, temporal or developmental differences. This is a considerable limitation for studying processes that occur in very specific cell types and/or over very short periods of developmental time (i.e., hours to days) where ‘capturing the moment’ is crucially important. The outcome from either scenario is that data derived from multiple cell types or developmental transitions can be blurred, a limiting factor for biological interpretation. However, these issues could be largely overcome by reducing the biological variation introduced by sample size and/or complexity, and by maintaining a high sensitivity of analysis.

**FIGURE 1:**
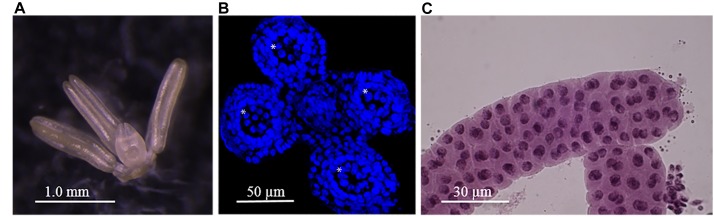
Barley anthers and meiocytes. **(A)** Mature barley anthers at late meiosis stage. **(B)** Anther’s Cross-section showing 4 pollen sacs (asterisk). **(C)** Meiocytes at zygotene stage within pollen sac after an anther squash.

Meiosis in barley cv. Golden Promise routinely occurs at around 6–7 weeks after planting when anthers from single spikelet sampled from around the middle of the developing inflorescence are generally 0.5–0.8 mm in length (Figure 1A). Sectioning, staining and cell counting revealed that approximately 10% of cells in the developing barley anther are meiocytes (Figure 1B), cells that undergo meiosis and subsequently develop into the haploid pollen grains that contain recombined parental chromosomes. By counting cells from successive 20 μm anther cross sections we estimate that each 0.6–0.8 mm anther contains approximately 7,000–9,000 cells. The meiocytes are larger than the surrounding cells (Figure 1B) (Colas et al., 2016) and are held within a sac like structure (pollen sac) that can be extruded by manual disruption and careful manipulation under the microscope (Figure 1C). Each single spikelet contains three developmentally synchronized anthers (Figure 1A). Here we have restricted our analysis to one, two or five whole developing anthers from the same spikelet or opposing spikelets either side of the rachis. As our previous results have shown a strong correlation between anther length and stage of meiosis we anticipated that anthers of 0.6–0.8 mm would be in the early stages of meiosis, which was confirmed by squashing and staining. From each spikelet, one of the three anthers was sacrificed and stained with acetocarmine. We concentrated on collecting anthers from triplets that were visually scored as being in early prophase I (leptotene and zygotene), a highly dynamic stage when the actual process of recombination is likely taking place (Figure 1C) (Barakate et al., 2014; Colas et al., 2016). This sampling and staging strategy confirmed that barley cv. Golden Promise anthers in early prophase I are generally within the 0.6–0.8 mm size range. We isolated sufficient staged material for multiple biological replicates comprised of one, two or five anthers to develop and test a pipeline for efficient analysis of the barley anther proteome.

### Development of the Micro-Proteomic Pipeline

We evaluated three strategies for proteomic sample preparation: SP3 (Single-Pot Solid-Phase-enhanced Sample Preparation; Hughes et al., 2012), HILIC (Hydrophilic Interaction Liquid Chromatography; Alpert, 1990), and 1D SDS/PAGE gel fractionation (Granvogl et al., 2007). The first two approaches are especially suitable for proteomic analysis of quantity-limited biological samples as they reduce losses of material by using a single reaction pot for all steps of sample preparation (lysis, reduction, alkylation, and trypsin digestion). One-dimensional gel fractionation is not as widely used for micro-scale proteomic analysis but does benefit from both simplicity and ease of removal of contaminants or/and detergents from the sample prior to mass spectrometry analysis.

For the evaluation of proteomic sample preparation strategies, three individual staged barley anthers were dissected from the same floret and placed directly into a fresh micro centrifuge tube containing either an extraction buffer (for SP3 and HILIC method) or LDS NuPage buffer (for gel fractionation method). All three samples were processed in parallel, each according to one of the three different protocols described in Section “Materials and Methods.” Samples were analyzed by Nano Liquid chromatography-tandem mass spectrometry (nLC-MS/MS) using an LTQ-Velos Orbitrap mass spectrometer and the corresponding proteins identified using MaxQuant software against the Uniprot Hordeum vulgare database (March 2017). The number of proteins identified with a minimum of two peptides in each of the samples were compared. There were 21 proteins identified using the SP3 strategy, 937 proteins identified using HILIC protocol and 1,279 proteins identified using the 1D SDS/PAGE gel fractionation method. As the last strategy was the most efficient in our hands, we chose to further explore this approach as our method of choice.

We first asked whether different amounts of starting material (i.e., different numbers of individual barley anthers) influenced protein coverage. For sample preparation, we used either one, two or five anthers with three biological replicates. To decrease variability of the samples we followed the routine: if two anthers were pooled, they were dissected from the same floret; when pooling five anthers, two were harvested from the same floret as the staged anther and another three from the closest opposing floret. Extracted protein samples were size-separated by NuPAGE 1-D gel electrophoresis (Supplementary Figure S1). Using alignment with protein molecular weight marker, the regions corresponding to 3–17, 17–62, and 62–250 kDa were cut out from the individual gel lanes and subjected to in-gel digestion with Trypsin. The resulting peptides were then sequenced using an LTQ-Velos Orbitrap mass spectrometer and the corresponding proteins identified using MaxQuant software against the Uniprot Hordeum vulgare database (March 2017). Using a minimum of two assigned peptides we identified 2,877 proteins from a single anther sample, 4,054 proteins from the sample of two pooled anthers and 4,307 proteins from the sample of five pooled anthers (Supplementary Table S1). We compared results obtained from different samples (one, two, or five anthers) with regards to the number of identified peptides, number of identified proteins and the percentage of MS spectra matched to the Uniprot Hordeum vulgare database (Table 1).

**Table 1:** Comparison of MS/MS results obtained by using one, two or five pooled barley anthers.

	1 Anther	2 Anthers	5 Anthers
No of identified proteins	3,410	4,699	5,033
No of proteins identified with 2 or more peptides	2,877	4,054	4,307
No of identified peptides	21,404	31,375	34,092
% of MS spectra matching Uniprot database (March 2017)	30.70%	39.20%	39.75%


**FIGURE 2:**
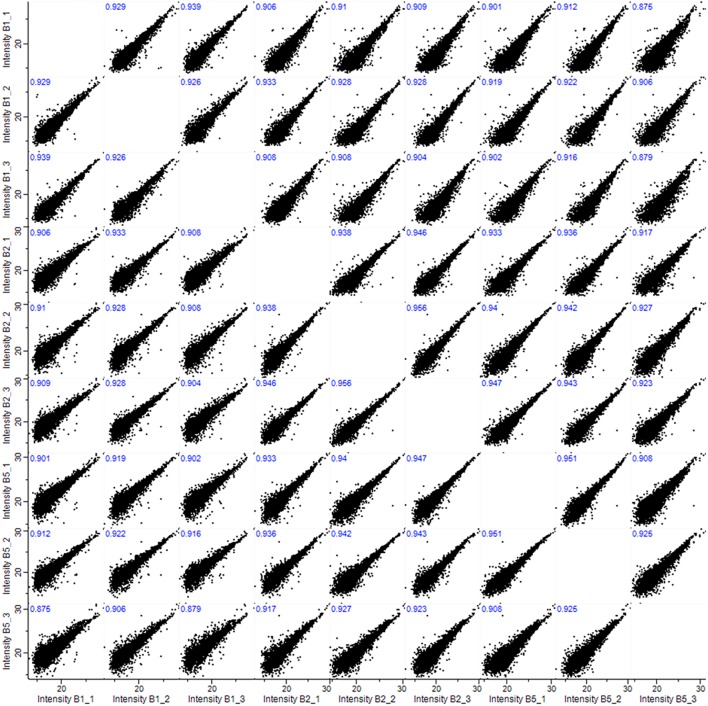
Pearson correlations (*R*^2^) among biological triplicates (1, 2, and 3) and among samples prepared from 1, 2, or 5 anthers (B1, B2, and B5).

All samples showed high inter-sample correlation coefficients (*R*^2^) among biological triplicates (*R* > 0.908) as well as among samples prepared from different numbers of anthers (*R* > 0.879), illustrating the superior reproducibility of the method (Figure 2). Our data demonstrate some advantage of using five pooled anthers over a single anther to maximize the number of identified proteins. However, five anthers, originating from two different florets, represent greater developmental variability and potentially meiotic asynchrony, than two paired anthers harvested from a single floret. Therefore, we based our final micro-proteomics workflow on two barley anthers from the same spikelet, with the third used for verification of the meiotic developmental stage by acetocarmine staining.

### The Proteome of Barley Anthers in the Early Stages of Meiosis

**FIGURE 3:**
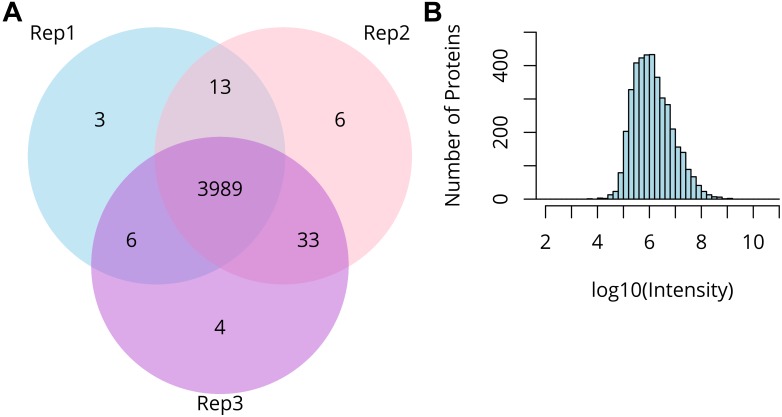
Summary of micro-proteomics results. **(A)** Venn diagram comparing proteins identified from three biological replicates of two pooled barley anthers. **(B)** Distribution of the protein intensities in the sample of two pooled anthers.

We used our micro-proteomic approach to analyze the proteome of barley anthers in the early stages of prophase I. The proteomic samples consisted of three replicates of two anthers, each originating from the same floret, with the third in each case used to assess the stage of meiosis. More than 31,000 peptides representing 4,699 proteins (with 4,054 proteins identified with 2 or more peptides) were identified. The protein landscape of paired barley anthers in triplicates showed >95% overlap in identified proteins, representing a dynamic range of expression levels across more than five orders of magnitude (Figure 3).

We next compared these results to a macro-proteomics study using seven replicates of ∼140 pooled barley anthers per biological replicate in the early stages of meiosis. The macro-proteomics identified a similar number of proteins: 4,738 proteins in total, with 4,111 identified with two or more peptides (Supplementary Table S2). There is a substantial overlap between proteins identified using micro- and macro-proteomics pipelines (2,912 proteins), with a group of 1,142 proteins exclusive to the micro-proteomic approach, and 1,198 proteins exclusive to the macro-proteomic approach. We inspected those two exclusive sub-sets of proteins and found some major differences in the Gene Ontology enrichment. The micro-proteomic specific proteome is significantly enriched for proteins assigned to the nucleus (126 proteins), chromosome (37 proteins), and protein-DNA complex (20 proteins). The sub-set exclusive to macro-proteomics shows slightly different distribution pattern with more proteins assigned to plastid (49) and Golgi apparatus (23).

Those differences between the two approaches could be explained by two features: first, we used a different methodology for sample lysis and preparation, with the micro-proteomics workflow being much simpler and thus reducing material losses, and second, the anthers collected for macro-proteomics included a ‘meiocyte enrichment’ step and represented a broader range of developmental stages which could in practice result in a more complex proteome.

### Subcellular Distribution and Functional Characterization of the Anther Proteome

We then classified anther proteins identified with two or more peptides by Gene Ontology (GO) terms in two domains: cellular component and molecular function. Only about three-quarters of the proteins were associated with one or more GO terms (3,148 out of 4,054), probably reflecting the incomplete state of barley genome/proteome annotation.

**FIGURE 4:**
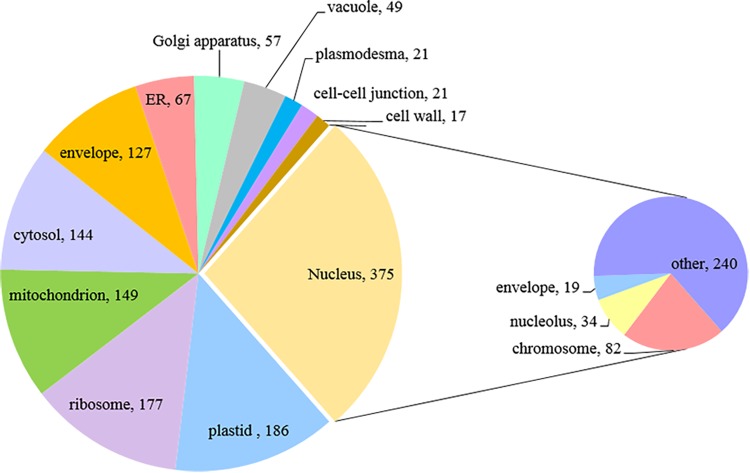
Subcellular localization of paired barley anther proteins predicted by GO annotation (Singular Enrichment Analysis, SEA).

For a global overview of the subcellular localization of identified proteins we performed Gene Ontology (GO) annotation based on cellular component, using an online tool AgriGO. The result showed the distribution pattern of 3,148 anther proteins, with the largest proportion assigned to the nucleus (378 proteins), followed by plastid (186), ribosome (177) and mitochondrion (149) (Figure 4). Interestingly, within the group of nuclear proteins, we found 82 chromosome-associated proteins. This group is represented by various proteins, including: histones (H1, H1.2, H2A, H2B, H3, and H4), proteins related to chromatin structure and remodeling (e.g., SMC1, SMS2, and SMC3), all six DNA helicases comprising Minichromosome Maintenance (MCM) Protein complex (MCM2-7) and some meiotic proteins (RFC1, RPA1, and MRE11).

**FIGURE 5:**
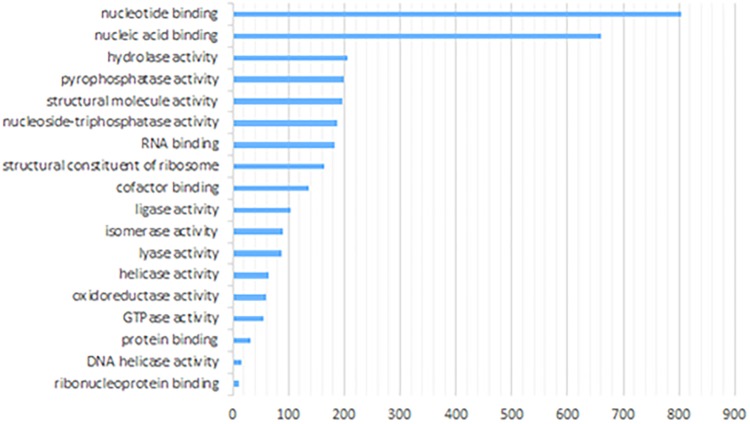
Enriched molecular functions of paired barley anther proteins predicted by GO annotation (Singular Enrichment Analysis, SEA).

We also performed functional enrichment analysis of anther proteome using the same online tool. Ten mostly enriched (*P* < 1.0E-0.2) molecular functions included nucleotide binding as the top category, followed by nucleic acid binding, hydrolase activity, pyrophosphatase activity, RNA binding and others (Figure 5). Notably, one of the overrepresented functional processes was DNA helicase activity, characteristic of enzymes that can bind and remodel nucleic acids. Some of those types of enzymes are essential players in regulating meiosis and recombination (Knoll and Puchta, 2011). Indeed, within this group comprising 16 proteins, we identified six proteins known from previous studies to be involved in meiosis (Ku70, Ku80, XPB2, SGS1, MCM7, and XPD). Results of additional Biological Process enrichment analysis are available as the (Supplementary Table S3).

### Detection of Meiosis-Associated Proteins

0.6 mm barley anthers contain developing meiocytes in the early stages of prophase I (leptotene, as estimated by acetocarmine staging) but they comprise only approximately 10% or less of anther tissue. Thus, we anticipated that meiosis specific proteins would exhibit relatively low-abundance in the whole anther proteome due to the dilution effect of other surrounding tissues. Nevertheless, our micro-proteomic pipeline was sensitive enough to detect several known meiotic proteins. By combining available information from barley Uniprot database, together with Blast2GO analysis, including functional annotation and InterPro scan^4^ we identified 57 known and putative meiotic proteins (Table 2), representing every step and process of early meiosis, i.e., chromosome condensation, synapsis, DNA double-strand break repair and crossover (CO) formation.

**Table 2:** Meiosis –related proteins identified in the paired anther barley proteome.

Barley MLOC^a^	Horvu number^b^	Gene name	Uniprot	Meiotic process	NP^c^	*Q*-value^d^
Meiosis genes			ID			
MLOC_12052	HORVU5Hr1G076340	ASY1	K9LWD7	Synapsis and chiasma assembly.	2	0.0022551
MLOC_55341.3	HORVU7Hr1G055440	AtRBR1	M0WWY5	Progression of the cell cycle from G1 to S phase; synapsis.	6	0
MLOC_74926	HORVU1Hr1G019590	AtSUN1/AtSUN2	F2E1M6	Meiotic attachment of telomere to nuclear envelope.	7	0
MLOC_37030	HORVU3Hr1G037960	AtSUN1/AtSUN2	M0VT65	Meiotic attachment of telomere to nuclear envelope.	10	0
MLOC_21019	HORVU4Hr1G067810	BUB3	M0W8G3	Spindle assembly checkpoint control.	2	0
MLOC_17320	HORVU4Hr1G023520	CDC45	M0VZ53	DNA replication initiation, double-strand break repair via break-induced replication, regulation of chromatin silencing at telomere.	2	0
MLOC_60707	HORVU6Hr1G013680	CDC2	M0WEB8	Cell cycle control.	6	0
MLOC_58535	HORVU2Hr1G049320	CHD4	M0XBC0	Chromatin organization.	2	0.0036714
MLOC_13176.1	HORVU1Hr1G094530	DDB1	M0UQM5	DNA repair.	35	0
MLOC_64588	HORVU4Hr1G008870	DDM1A	M0Y064	ATP-dependent DNA helicase that plays a role in formation, organization, stability and heritability of heterochromatin.	19	0
MLOC_22034.1	HORVU3Hr1G066930	FVE	M0VBU6	Chromatin organization, DNA repair.	5	0
MLOC_52096.4	HORVU2Hr1G058940	HEN1	M0WJD3	Small RNA 2′-O-methyltransferase; HEN1 homolog in human involved in gametogenesis.	7	0
MLOC_10230	HORVU6Hr1G061010	HOP2 (Hsp70–Hsp90 organizing protein 2)	M0UEM7	Mediates the association of the molecular chaperones HSP70 and HSP90.	31	0
MLOC_54143.1	HORVU4Hr1G007340	HOP2 (Homologous-pairing protein 2 homolog)	M0WS23	Bivalent formation and segregation of homologous chromosomes in meiosis.	4	0
MLOC_45046	HORVU4Hr1G059390	HSP70	M0X093	Required for pachytene progression.	32	0
MLOC_72488	HORVU5Hr1G072420	HSP90	Q7XJ80	Required for pachytene progression.	63	0
MLOC_62977	HORVU5Hr1G012090	KU70	G3K4H8	DNA non-homologous end joining (NHEJ) required for double-strand break repair.	13	0
MLOC_49689	HORVU0Hr1G038620	Ku80	F2DIX5	DNA non-homologous end joining (NHEJ) required for double-strand break repair.	26	0
MLOC_78807.1	HORVU1Hr1G044350	LIG1	M0Z870	Repair of both single strand breaks (SSBs) and double strand breaks (DSBs).	21	0
MLOC_63534	HORVU3Hr1G081720	MAD1	M0XVV2	Anaphase promoting complex.	2	0
MLOC_36301	HORVU5Hr1G028260	MCM7	M0VQ00	DNA helicase, DNA replication.	34	0
MLOC_63119	HORVU5Hr1G053350	MND1	M0XUD5	Chromosome pairing and double-strand break repair.	2	0
MLOC_36970	HORVU2Hr1G031130	MEL1	F2EF16	Chromosome condensation.	46	0
MLOC_64638	HORVU3Hr1G037080	MOS4/BCAS2	M0Y0E4	Required for meiosis prophase I in mouse spermatogenic cells; regulates mRNA splicing of functional genes.	5	0
MLOC_80996	HORVU2Hr1G116540	MRE11	F2DMY1	DNA double-strand break repair.	11	0
MLOC_43931	HORVU1Hr1G030930	Msh2	F2EIF8	Component of the post-replicative DNA mismatch repair system (MMR).	11	0
MLOC_38120	HORVU2Hr1G085940	Msh3	M0XDS1	Component of the post-replicative DNA mismatch repair system (MMR).	6	0
MLOC_59094	HORVU6Hr1G064940	MTA	M0XDG6	In yeasts essential for meiosis and sporulation.	2	0
MLOC_5045	HORVU1Hr1G043110	NBS1	F2CVB7	DNA double-strand break repair (part MRN complex); functions in early stages of meiosis.	5	0
MLOC_11755	HORVU3Hr1G074660	NIH	M0UKF1	Mitotic to meiotic cell cycle switching.	17	0
MLOC_9899	HORVU2Hr1G082720	PCH2	M0ZFK3	Synapsis, DNA double-strand break formation.	5	0
MLOC_2310	HORVU6Hr1G088120	PCNA1/2	M0VD44	Regulation of DNA replication; mismatch repair.	16	0
MLOC_80839	HORVU7Hr1G042100	RAD23A	F2DUJ6	Nucleotide excision repair.	12	0
MLOC_61890	HORVU5Hr1G020580	RAD50	M0XPE7	Double-strand breaks (DSBs) repair by non-homologous end joining (NHEJ).	6	0
MLOC_67983.1	HORVU3Hr1G081760	RAD52-1	Q9LEH5	Double-strand break repair via homologous recombination.	8	0
MLOC_38431	HORVU5Hr1G078090	RAD52-2	F2CYC4	Double-stranded DNA break repair via homologous recombination.	3	0
MLOC_26026	HORVU5Hr1G122680	RAD52-2	F2CR01	Double-stranded DNA break repair via homologous recombination.	5	0
MLOC_9867.1	HORVU2Hr1G109910	RCC2	M0ZFG6	Chromosome condensation.	7	0
MLOC_20390.2	HORVU0Hr1G002110	RECQ4B	M0V8N1	Double-strand break repair via homologous recombination.	9	0
MLOC_20390	HORVU0Hr1G002100	RECQL3_b	M0V8M7	Double-strand break repair via homologous recombination.	9	0
MLOC_51067	HORVU7Hr1G091790	RFC1	M0WFN2	Meiotic recombination via a specific pathway for crossovers (COs) that involves the formation of double Holliday Junction (dHJ) intermediates.	25	0
MLOC_17360.1	HORVU6Hr1G078830	RFC5	F2DRS5	Chromatin assembly and remodeling, DNA repair.	13	0
MLOC_19104	HORVU6Hr1G081140	RPA1A	M0V669	Later stages of meiotic recombination events, formation of class I crossovers; chiasma assembly.	3	0
MLOC_81884	HORVU6Hr1G094080	RPA2A	F2EJA7	DNA replication, recombination and repair.	9	0
MLOC_45783.1	HORVU3Hr1G034860	RPA3B	F2D0W0	DNA replication, recombination and repair.	2	0
MLOC_11443	HORVU2Hr1G066910	SAD2	F2DY16	Nuclear transport receptor.	16	0
MLOC_4342	HORVU2Hr1G064000	SCC2	F2CVK4	Meiotic sister chromatin cohesion.	4	0
MLOC_5703.1	HORVU4Hr1G031480	SCC3	F2DZC8	Cohesion of sister chromatids after DNA replication.	19	0
MLOC_37758	HORVU6Hr1G051930	SET	F2DD97	Double-strand break repair via homologous recombination.	11	0
MLOC_70481	HORVU7Hr1G002220	SKP1	Q9M3X1, F2D7S9	Correct chromosome segregation during tetrad formation.	9, 9	0, 0
MLOC_57784.1	HORVU7Hr1G066300	SMC1	M0X823	Central component of cohesin, chromosome cohesion.	19	0
MLOC_4644.1	HORVU3Hr1G088700	SMC2	M0WAH1, F2E3E6	Chromosome organization and segregation.	25, 29	0, 0
MLOC_54939.1	HORVU1Hr1G093520	SMC3	M0WV69, M0UNZ2	Chromosome segregation.	14, 10	0, 0
MLOC_10713	HORVU3Hr1G054730	SUMO1	M0UGE2	Double-strand break repair.	4	0.0095319
MLOC_38181	HORVU6Hr1G067930	TOP2A	M0VXE2	Resolution of meiotic recombination intermediates; sister chromatid segregation.	19	0
MLOC_66388.3	HORVU1Hr1G019340	XPD	M0Y774	Conducts nucleotide excision repair (NER).	5	0
MLOC_49048.2	HORVU3Hr1G064300	XPB2	M0WCV8	Conducts nucleotide excision repair (NER).	2	0


*^*a*^Whole-genome shotgun (WGS) barley reference sequence database (The International Barley Genome Sequencing Consortium [IBSC], 2012) accession numbers (MLOC) for barley genes encoding identified meiosis-related proteins.*

*^*b*^Updated Ensembl Plants database (Mascher et al., 2017) accession numbers (HORVU) for barley genes encoding identified meiosis-related proteins.*

*^*c*^The number of matched peptides.*

*^*d*^Q-value (the ratio of reverse to forward protein groups) generated by MaxQuant software. It operates as a p-value, where smaller is more significant and reflects better match quality.*

Amongst the detected proteins there are some with well-established meiotic functions: two proteins essential for chromosome condensation; a homolog of a protein argonaute (MEL1) and a homolog of Regulator of Chromosome Condensation 2 (RCC2); proteins involved in key later events of meiosis: Asynapsis 1 (ASY1) essential for synapsis and a homolog of Meiotic Recombination 11 (MRE11) involved in the DNA double-strand break resection; a Replication Factor C subunit (RFC5) homolog that is crucial for CO formation and proteins SMC2 and SMC3 with functions in chromosome segregation (Mercier et al., 2014).

**FIGURE 6:**
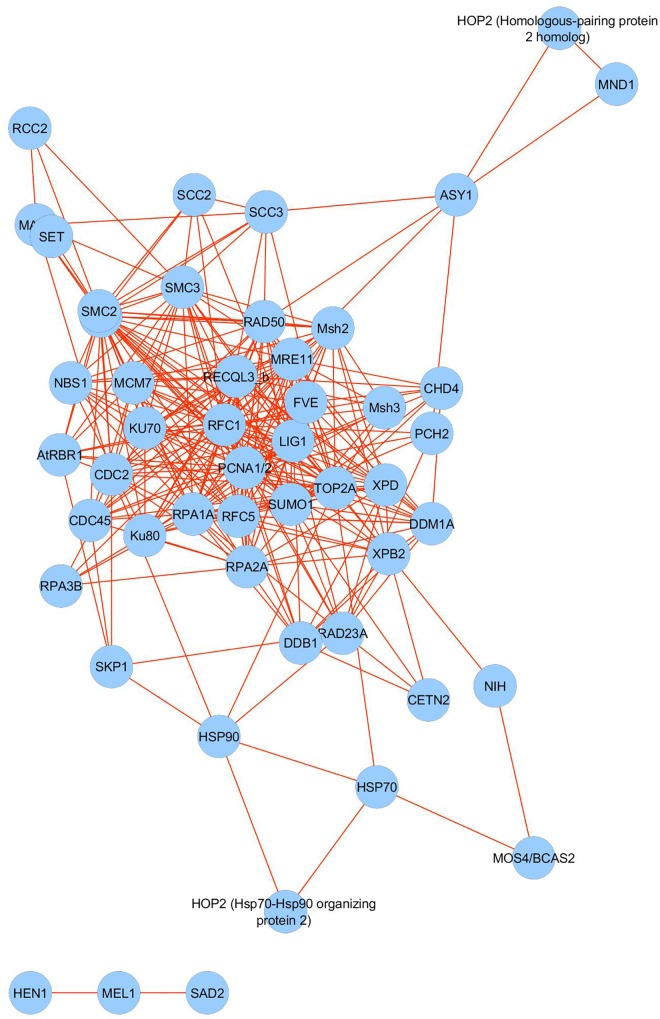
Interaction network analysis of 57 meiosis-associated proteins identified in the barley anther proteome.

We searched the barley gene identifiers (MLOC numbers) for 57 meiosis-related proteins detected in the micro-proteomics dataset against the STRING database version 10.5 (Szklarczyk et al., 2015) for protein–protein interactions. We focused only on interactions between proteins belonging to the meiotic dataset and selected only those that had a confidence score ≥ 0.4 (medium confidence). ‘Confidence score’ defines interaction confidence and is computed by integrating the probabilities of protein–protein interactions from various types of evidence. The resulting network of interactions between meiosis-related proteins had 56 nodes and 303 interactions (PPI enrichment *p*-value: <1.0e-16) (Figure 6). It shows significantly higher connectivity than a random set of proteins of similar size, taken from the genome; namely more than 5 interactions per node, whereas the random expectation would be approximately one. Our data therefore suggest that these 57 meiosis-associated proteins forming the network, are biologically and functionally connected.

### Micro- vs. Macro-Proteomics

To assess how representative the proteome derived from our micro-proteomic pipeline was, we compared the number of identified meiotic proteins to that obtained using a macro-proteomics approach (seven replicates of ∼140 anthers each). Surprisingly, our macro-proteomics detected a comparable number of meiosis-associated proteins (61 proteins). Meiotic proteins identified by macro-proteomics but not by the micro-proteomics pipeline included DMC1, DSS1, FANCM (the latter identified only by one peptide in the macro-proteomics dataset), Msh6 and PRD1. Interestingly, micro-proteomics dataset also contained 4 meiosis-related proteins that were not identified using macro-proteomics approach, namely BUB3, responsible for spindle assembly checkpoint control; CHD4 involved in chromatin condensation; SCC2 functioning in sister chromatid cohesion and XPB2, protein that conducts nucleotide excision repair (Mercier et al., 2014).

We anticipate that this variation is likely due to the different characteristics of the samples. In the case of the macro-proteomics experiment, each sample consisted of more than 140 pooled anthers (in the size range 0.6–0.8 mm) and represented a wider range of developmental stages of meiosis and likely a wider scope of proteins. In the two precisely staged and developmentally synchronized anthers, we observe slightly fewer meiotic proteins but those that are detected are more likely to be stage specific. Another factor contributing to the discrepancy may be the limited amount of total protein used in the micro-proteomics approach, hindering detection of very low abundance proteins. However, approximately the same number of unique proteins was identified in each study.

## Discussion

The motivation behind the current study was our desire to explore the proteinaceous machinery involved in meiosis in the cereal crop species barley, in which the process of genetic exchange (recombination) happens within a short developmental time frame and occurs near the telomeric ends of its seven large metacentric chromosomes (The International Barley Genome Sequencing Consortium [IBSC], 2012). Our goal is to understand why recombination is predominantly telomeric and whether it may be possible to manipulate this feature by altering the function or timing of the appearance of key players in the recombination machinery. Our main strategy is to use mutants in key meiotic proteins that based on cytological or genetic evidence reveal perturbations in the frequency or pattern of recombination (Barakate et al., 2014; Colas et al., 2016). As a forerunner to investigating such effects we considered it essential to develop a robust and reliable protocol for investigating the proteome of cells and tissues that we could confidently assign to a specific meiotic stage. In our case, each barley spikelet contains three developmentally synchronized anthers providing an opportunity to accurately and destructively stage one while preserving the other two for molecular analysis. The main issue we then had to solve was how to reliably scale methods for plant proteomics for single or two anthers of 0.6–0.8 mm in length to comprehensively reveal the genome-wide proteome.

Recent rapid advances in proteomics technologies have significantly increased the depth of the protein coverage in macroscale studies, however, the analysis of microscale samples is still challenging, especially in the case of plant tissues. We considered sensitivity as being crucial, especially as only about 10% of the cells in whole barley anthers are the ones that undergo meiosis. Any useful approach must therefore be capable of detecting low abundance proteins from what remains a relatively complex mix of different cell types. As meiosis is well described in several species it is possible to test both representation and sensitivity by identification of key meiotic proteins in the anther proteome through protein or DNA sequence homology. Here we developed and describe a robust micro-proteomics workflow for the analysis of individual or paired barley anthers, that could be adapted to other tissue-limited plant samples. The workflow detects more than 4,000 proteins from these small amounts of material, covering a dynamic range of protein expression levels across five orders of magnitude. We consistently identified ∼2,800 proteins in a single 0.6 mm anther and ∼4,000 proteins in a paired-anther sample. We believe this demonstrates an important technological advance in plant proteomics given the limited amount of starting material.

We deployed our micro-proteomics workflow to investigate the proteome of the developing barley anther containing pollen mother cells in the early stages of meiosis. We successfully identified several meiosis-related proteins, proving that the approach is highly sensitive and powerful. To date, no work has been published previously on proteomics of precisely staged individual plant anthers. Published proteomics studies are typically large-scale, reporting analyses of samples consisting of large numbers of pooled anthers. For example, about 10,000 RAMs, developing Rice Anthers around the time of Meiosis, have been used for high-resolution mass spectrometry-based proteomic and phosphoproteomic analyses resulting in the identification of 4,984 proteins and 3,203 phosphoproteins (Ye et al., 2015). A similar experiment using 10,000 RAMs for lysine acetylation analysis concluded in the identification of 1,354 lysine acetylation sites in 676 proteins (Li et al., 2018). A comparable number of developing Arabidopsis anthers at stages 4–7 (corresponding to pre-meiotic and meiotic stage) and 8–12 (anthers before dehiscence), has been used to investigate the proteome and phosphoproteome, producing a dataset of 3,908 phosphorylation sites (Ye et al., 2016). Each of these studies analyzed protein extracts obtained from millions of different cells, from anthers representing a wide range of developmental stages.

While the whole anther proteome has been studied previously (Sheoran et al., 2009; Uváèková et al., 2012; Liu et al., 2015; Ye et al., 2015, 2016) it is generally the identification of proteins involved in meiosis and recombination that have been sought to provide insight into the molecular mechanism of plant sexual reproduction. We used precisely staged, paired barley anthers to identify 57 known and putative meiotic proteins that represent many steps and processes of early meiosis, i.e., MEL1 and RCC2 homologs essential for chromosome condensation; ASY1 protein involved in synapsis and a homolog of MRE11 involved in the double-strand break repair. Interestingly, we also identified several proteins that have not yet been proven to be involved in meiosis in plants, but which are known to participate in meiosis regulation in animals. For example, MOS4/BCAS2 which has multiple functions in several pathways including splicing and DNA damage repair, has been shown to be a critical factor for the initiation of meiosis prophase I in male mouse germ cells (Liu et al., 2017). Similarly, we identified an N6-adenosine-methyltrasnferase MT-A70-like protein (MTA), which methylates adenosine residues of some mRNAs thus modulating their stability, transport, splicing and/or translation (Zhong et al., 2008). Recently, several studies have reported a strong link between RNA methylation and meiosis in yeasts and *Drosophila*, with MTA homologs being important regulators of this process (Hongay and Orr-Weaver, 2011; Schwartz et al., 2013).

In pooled rice meiocytes in prophase I, Collado-Romero et al. (2014) reported the identification of 167 proteins belonging to different functional groups that could potentially be involved in the early meiotic processes. Selection of these proteins was based on their putative function in chromatin structure and remodeling, nucleic acid binding, cell-cycle regulation and cytoskeleton. The study provided interesting examples of candidate proteins that might be meiosis-related; however, despite the arduous sampling and pooling of similarly staged meiocytes it failed to identify key proteins known to be involved in the early stages of meiosis (e.g., RCC2 or ASY1).

More recent proteomics and phosphoproteomics analysis of pooled developing rice anthers around the time of meiosis (RAM) resulted in the detection of 35 meiosis-related proteins, including those of well-confirmed meiotic function, e.g., MEL1, PAIR2, and PAIR3 or MER11 (Ye et al., 2015). Comparison of this protein group with the set of 57 meiotic proteins identified here, revealed 18 identical meiotic proteins in both datasets: MEL1, MRE11, PAIR2 (ASY1), RPA2C, RFC5, RCC2, SMC3, SCC3, SMC1, RAD50, LIG1, RPA1, RFC1, SKP1, RBR1, MPA1, RAD23, BUB3 and kinesin (we did not consider the latter a strictly meiotic protein so it was not included in our list of 57 meiosis-related proteins, however, it was detected within the barley paired anther proteome). There were also proteins that were exclusive to the Ye et al. (2015) dataset or specific to our study. We anticipate that this variability in detected meiotic proteins is likely due to the different nature and complexity of the biological materials used: a few thousand rice RAMs covering a wide range of meiotic stages in Ye et al. (2015) versus two highly synchronized and precisely staged barley anthers in our analysis.

Our micro-proteomics workflow provides an exciting opportunity to investigate the protein machinery regulating specific stages of meiosis, allowing us to generate the first successful label-free genome wide proteomic analysis of individual staged barley anthers. We presume that the success of the method lies in the reduced number of lysis and protein precipitation steps, which lowers the possibility of sample loss and extraction biases. However, our workflow is not a typical ‘single reaction tube’ (SRT) protocol, as it employs 1D-SDS/PAGE gel fractionation to reduce sample complexity and subsequent multi-step ‘in gel ‘trypsin digestion of proteins. Interestingly, none of the classical SRT protocols we have tried (SP3 and HILIC) turned out to be applicable for single or paired anther proteome analysis in our hands. SRT approaches have been successfully used to analyze suboptimal animal samples and an SRT method employing magnetic bead-based technology (SP3) has proven to be efficient for the characterisation of single human oocyte proteome (Virant-Klun et al., 2016). Another study used a modified version of the ‘one pot’ method coupled with iterative data analysis to analyze *C. elegans* proteome at the single worm level (Bensaddek et al., 2016). The lack of published studies on using SRT in plant microscale sample proteomics raises the question of its current relevance in plant proteomics research.

The new micro-proteomics approach identified a comparable number of total proteins to the macro-proteomics dataset that we obtained by analysis of ∼1,000 pooled barley anthers. Both datasets contain more than 4,000 proteins, covering a dynamic range of expression levels across five orders of magnitude. This result demonstrates that scaling down the amount of starting material doesn’t necessarily have a negative effect on the sensitivity and efficiency of the micro-proteomic approach. When we compared datasets produced by macro- and micro-proteomics we found 2,912 overlapping proteins and 1,142 proteins exclusive to micro-proteomics approach, together with 1,198 proteins specific to macro-proteomics method. Closer examination of those two exclusive subsets revealed differences in the Gene Ontology (GE) annotation enrichment. Namely, the distribution pattern of proteins specific to micro-proteomics approach shows 126 proteins assigned to the nucleus, including 37 chromosome-associated proteins. Conversely, in the subset of proteins unique to macro-proteomics approach, we could not find any proteins with predicted nuclear localisation but there was a small proportion assigned to cellular compartments not represented in the micro-proteomics subset, like Golgi apparatus. This finding suggests various resolutions of macro- and micro-proteomics approaches and points out the latter might be more suitable method for analysis of low-abundant nuclear proteins.

We report the first insight into the proteome of barley anthers in the early stages of meiosis performed at the individual (paired) anther level. The wider implication of our work is that it should be suitable for proteomic examination of many size-limited plant specimens. The relative advantage of micro-proteomic over the macro-proteomic workflow lies in its simplicity, speed and a biological relevance. For us, the ability to assess the proteome of individual and precisely staged anthers opens the way to a better understanding of specific stages of meiosis, its regulation, and response to biotic and abiotic stimuli. The pipeline is sensitive enough to detect meiosis-associated proteins in the developing barley anther and importantly it performs as well as previously used macro-proteomics approach. We are currently applying the approach to explore dynamic changes occurring in the barley anther proteome over developmental time and to interrogate dysregulated cellular networks induced by developmental mutations and environmental change that cause both semi-sterility and changes in the pattern of recombination. In the future, we plan to use the micro-proteomics pipeline to investigate the proteome of individual populations of meiocytes (e.g., single pollen sacs inside anther’s locules – Figure 1), thus reducing the background information from non-meiotic tissues of an anther. When used in combination with constantly improving mass spectrometer’s sensitivity, it has a potential of becoming an advanced tool to study plant meiosis.

## Data Availability

The datasets generated for this study can be found in the ProteomeXchange with the accession number PXD010887.

## Author Contributions

DL and RW initiated and designed the study. DL performed the experiments and analyzed the data. RZ carried out part of the bioinformatics analysis and generated some figures. NU maintained barley plants, collected barley anthers for proteomic analysis and performed some experiments. IC estimated an average number of meiocytes in the barley anther. DL and RW wrote the manuscript. RZ and IC critically reviewed the manuscript. All authors read and approved the final manuscript.

## Conflict of Interest Statement

The authors declare that the research was conducted in the absence of any commercial or financial relationships that could be construed as a potential conflict of interest.

## Footnotes

1 https://www.resynbio.com/product_hilic.htm

2 http://systemsbiology.cau.edu.cn/agriGOv2/index.php

3 https://string-db.org

4 https://www.blast2go.com/

## Supplementary Material

Figure S1 Single anther protein samples fractionated on NuPage 1-D gel, stained with InstantBlue; MW – Protein Standard (SeeBlue Plus2), molecular weights in kDa.

Table S1 Protein Groups output table from MaxQuant software containing all proteins identified in the micro-proteomic approach.

Table S2 Protein Groups output table from MaxQuant software containing all proteins identified in the macro-proteomics approach.

Table S3 Enriched Biological Process terms of paired barley anther proteins predicted by GO Annotation (Singular Enrichment Analysis, SEA).
