# Supplementary figures and images for: Application of a Sensitive and Reproducible Label-Free Proteomic Approach to Explore the Proteome of Individual Meiotic-Phase Barley Anthers
Source: Front Plant Sci. 2019 Apr 2;10:393. doi: 10.3389/fpls.2019.00393 (PMC6454111; doi:10.3389/fpls.2019.00393)

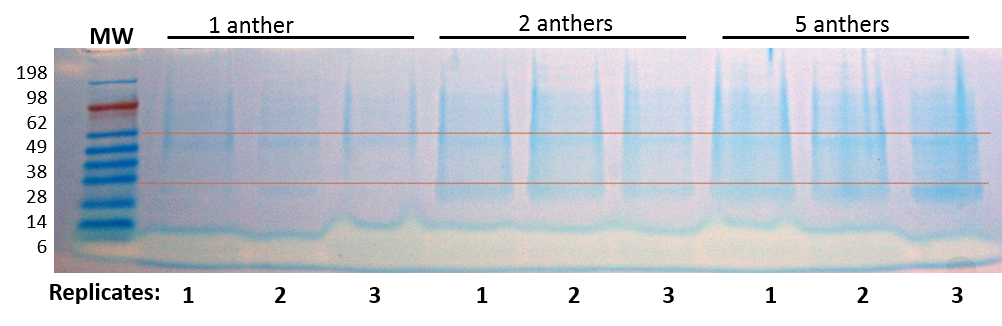

Supplement: Figure S1 — Single anther protein samples fractionated on NuPage 1-D gel, stained with InstantBlue; MW – Protein Standard (SeeBlue Plus2), molecular weights in kDa. [file Image_1.TIF]
